# A Comprehensive Literature Review for Total Hip Arthroplasty (THA): Part 2—Material Selection Criteria and Methods

**DOI:** 10.3390/jfb16050184

**Published:** 2025-05-18

**Authors:** Salvatore Garofalo, Chiara Morano, Luigi Bruno, Leonardo Pagnotta

**Affiliations:** Department of Mechanical, Energy and Management Engineering, University of Calabria, Via P. Bucci 44C, 87036 Rende, Italy; chiara.morano@unical.it (C.M.); luigi.bruno@unical.it (L.B.)

**Keywords:** total hip arthroplasty (THA), total hip replacement (THR), bearing surfaces, selection methods, material selection

## Abstract

Total Hip Arthroplasty (THA) is a widely used surgical procedure to restore mobility and reduce pain in patients with hip joint disorders. Implant success and longevity are influenced by the selection of appropriate materials. This study presents a comprehensive literature review based on structured searches in Scopus and Web of Science, focusing on material selection criteria and methods in THA. The inclusion criteria targeted original studies and reviews addressing material properties, selection techniques, and clinical performance. A bibliometric analysis and keyword co-occurrence network were used to highlight major research themes. The review examines traditional materials such as Metal-on-Polyethylene (MoP), as well as advanced options like ceramics, composites, and Functionally Graded Materials (FGMs). Key challenges discussed include aseptic loosening, wear resistance, and stress shielding. Selection methodologies such as Multi-Criteria Decision-Making (MCDM), Weighted Properties Methods (WPM), and computational tools like Ashby charts and CES Selector are analyzed. The findings from international arthroplasty registries show that more than half of implant failures are linked to material-related factors. This study therefore aims to guide material selection processes in THA by aligning clinical performance with biomechanical and biological requirements, supporting improved implant outcomes and long-term surgical success. Future developments should focus on patient-specific solutions and continuous innovation.

## 1. Introduction

Total Hip Arthroplasty (THA) is a complex and highly sophisticated surgical procedure designed to replace a damaged hip joint with an artificial prosthesis. The process begins with the removal of the compromised femoral head, which is replaced with an artificial spherical head. Additionally, the acetabulum, the socket in the pelvis that holds the femoral head, is replaced with a prosthetic cup. Inside this, an insert is placed to provide a smooth and durable surface, ensuring the flat and long-lasting movement of the femoral head.

THA is a common surgical intervention for end-stage Hip Osteoarthritis (OA), and it is an orthopedic surgery that can significantly improve the quality of life for patients suffering from debilitating hip joint conditions [1]. Specifically, OA is a prevalent degenerative joint disease, particularly among the elderly. A systematic review and meta-analysis reported that the radiographic prevalence of hip OA is highest in Europe, at 12.59%. In the United States, OA affects approximately 32.5 million adults [2,3]. According to the American Joint Replacement Registry (AJRR), over 4.3 million hip and knee arthroplasty procedures were recorded in the United States from 2012 to 2023. In Europe, countries like Germany and Switzerland report high rates of hip replacement surgeries, with Germany performing approximately 301 procedures per 100,000 population in 2021 [4,5]. The increasing global burden of OA and the rising demand for joint replacement surgeries necessitate the optimization of THA outcomes. Material selection plays a crucial role in implant longevity and performance.

The decision to proceed with a hip prosthesis is typically made after careful clinical evaluation and often when less invasive treatments are no longer effective. The primary goals of the surgery are to relieve pain, restore joint function, and improve the patient’s mobility [6].

The aging population, combined with increasingly reliable surgical techniques and a greater availability of logistical, instrumental, and human resources, has led to an exponential increase in the number of hip replacement surgeries performed annually worldwide. The growth in the number of such surgeries is alarming, prompting many researchers to study its evolution. A common goal is to accurately estimate the growth in volume to prevent public health systems from being caught unprepared and to allow for the proper planning of the investments needed to support the costs of new operating rooms, new technologies, artificial prostheses, and the workforce, including the training of surgeons.

Forecasting studies have covered almost the entire world [7], from the more forward-looking European countries [8,9,10,11] to the United States [12], Asian countries [13,14,15], and Australia [16]. All studies have confirmed that hip prostheses are currently among the most frequently performed surgical operations in the world, second only to knee prostheses, with only slight variation between countries.

Although little can be done to slow the growth in the number of primary surgeries—since they result from a series of largely uncontrollable factors related to degeneration, injury, or disease of the hip joint (such as OA, rheumatoid disease, osteonecrosis, hip fractures, hip dysplasia, and post-traumatic arthritis, often aggravated by obesity and age) [7,17]—much can be done to reduce the number of prosthesis repairs caused by the failure of previously implanted prostheses.

Studying the causes of prosthesis failure can not only facilitate their restoration but also provide a wealth of information that will help improve the surgery and extend the prosthesis’s lifespan. Currently, although there is insufficient information to determine the exact longevity of a hip replacement, data from arthroplasty registries suggest that approximately 75% of hip replacements last between 15 and 20 years. Additionally, over 50% of hip replacements endure for 25 years in patients with osteoarthritis [18]. Today, it is evident that the long-term durability of a hip replacement is influenced by various factors and their interaction. In addition to the quality of the implant itself, elements such as the diagnosis (the cause leading to surgery), patient characteristics (age, weight, and activity level), the surgeon’s skills and expertise, and the surgical technique play a crucial role in the implant’s longevity [19]. Despite initial positive outcomes, revision rates for hip prostheses—the need for a second surgery to replace or repair the original prosthesis—have steadily increased in recent years. This may be due to various factors, including implant wear, postoperative complications, or technical errors during the initial surgery [20].

As the field advanced, the importance of material selection in determining the success or failure of hip prostheses became increasingly clear. Data from the National Joint Registry (NJR) and other studies have shown that mechanical wear, aseptic loosening, and material degradation are common causes of prosthetic failure [21]. These failures underscore the critical role that material selection plays in ensuring the longevity and functionality of implants.

In recent years, advanced manufacturing techniques have revolutionized the development of biomedical devices, particularly through the implementation of Additive Manufacturing (AM). AM, commonly referred to as 3D printing, offers unprecedented design freedom, enabling the fabrication of complex and patient-specific structures with controlled porosity and internal architecture. This has led to significant advancements in tissue engineering, drug delivery systems, and bioresorbable implants [12,14,20]. Notably, materials such as poly(lactic acid) (PLA) and poly(ε-caprolactone) (PCL), which are frequently employed in Fused Deposition Modeling (FDM), have shown great promise due to their biodegradability and biocompatibility [17,19]. As the field continues to evolve, the integration of AM with smart polymers and functional biomaterials opens new avenues for the development of responsive and tailored therapeutic platforms [15,21].

Despite the significant advancements in biomaterials for THA, the literature lacks a consolidated framework that evaluates and compares the criteria and structured decision-making methods guiding material selection. Most existing reviews emphasize clinical outcomes or mechanical properties in isolation. To fill this gap, this review systematically examines both the criteria and selection methodologies employed in THA material choice, including the use of Multi-Criteria Decision-Making (MCDM) methods, such as the Analytic Hierarchy Process (AHP), and computational tools like CES Selector and Ashby charts, which are frequently used to evaluate and rank potential materials based on these factors [22,23]. The underlying hypothesis is that improved material selection, based on multi-criteria evaluation and patient-specific considerations, can contribute to reduced revision rates and enhanced long-term outcomes.

This study aims to review the current criteria and methods used in the selection of materials for THA components, identify their role in implant longevity and failure mechanisms, and evaluate emerging technologies aimed at enhancing implant performance and reducing revision rates. Specifically, the focus of this work is to identify the benefits and drawbacks of various types of biomaterials, analyzing their applications in hip prostheses. The review targets to equip the reader with the essential information needed to make conscious decisions about material choice in THA. Whether using traditional materials like metal-on-polyethylene or innovative approaches like Functionally Graded Materials (FGMs) and hybrid composites, the goal is to ensure that the chosen materials offer the best possible outcomes for patients [24,25]. Finally, it should be noted that this paper is the second part of a broader study: while Part 1 provides an in-depth comparative analysis of the most commonly used materials and biomaterials in THA—including their clinical performance, associated complication rates, and revision outcomes—Part 2 focuses specifically on the selection criteria and decision-making methods for choosing appropriate materials.

## 2. Literature Analysis and Methodology

**Figure 1:**
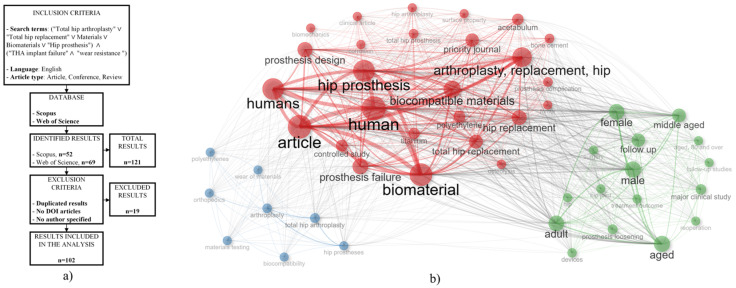
(**a**) Flow chart of the selection process of the bibliographic references; (**b**) keyword co-occurrence network based on the references analyzed.

The research strategy is schematically described in the flow chart shown in Figure 1. A series of keywords was used to define the bibliographic framework. After several attempts that involved properly combining different keywords with Boolean *AND/OR* operators, the final research string in the first block of Figure 1a) was identified. The most common scientific databases (Scopus and Web of Science), which are widely recognized as the most influential databases, yielded a total of 121 documents. After removing all duplicate documents, and those whose DOI and authors were missing, a final list of 102 unique documents was obtained. Afterward, a statistical analysis was performed. The analysis was facilitated through the utilization of Bibliometrix, an application developed by Aria and Cuccurullo [26]. To explore the conceptual structure of the THA research domain, a co-occurrence network (Figure 1b)) was generated using author keywords extracted from the analyzed literature dataset. The resulting graph visualizes the relationships between frequently co-occurring terms, offering insights into the thematic organization and research trends within the field. Each node in the network represents a unique keyword, with a node size proportional to the frequency of occurrence in the dataset. Edges (or links) indicate co-occurrence relationships—i.e., how often two keywords appear together in the same publication. Thicker edges reflect stronger co-occurrence. The network is divided into distinct clusters, each identified by a different color, suggesting the presence of research subfields or thematic areas. One prominent cluster centers around hip prosthesis, prosthesis failure, and replacement, indicating a strong thematic focus on surgical outcomes and postoperative issues. Another cluster groups terms such as biocompatibility and material testing, reflecting research concerned with the mechanical behavior and optimization of hip implants. A third cluster includes keywords like male, female, middle age, and follow up, highlighting the clinical and patient-centered aspects of THA.

This network reveals the interdisciplinary nature of THA research, combining elements from biomechanics, materials science, surgical technique, and clinical management. The clustering further emphasizes how different aspects of THA are interconnected in the scholarly literature.

Additionally, some of the existing reviews were used as an auxiliary search database to broaden the view to include further potentially relevant research. Moreover, in analyzing the data extracted from various national joint registries, WebPlotDigitizer [27] was employed to digitize graphical data, which were then processed and visualized using OriginLab [28] to create the graphs presented in the review paper.

## 3. Hemiarthroplasty and Total Hip Arthroplasty

The hip is crucial for human movement, as it supports the upper body’s weight during activities like standing, walking, and running. Its stability is vital for the effective execution of these functions.

**Figure 2:**
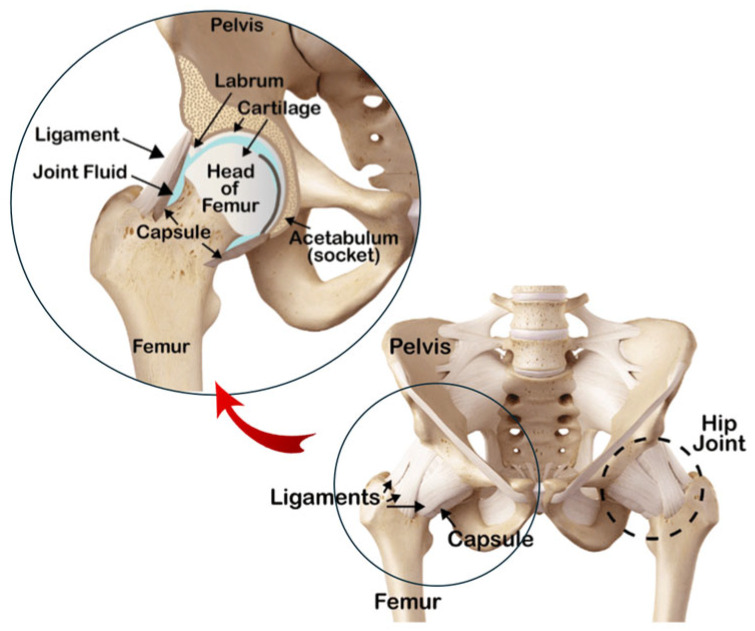
Hip anatomy, readapted from [29].

The hip joint connects the femur to the pelvis through the femoral head, which fits securely into the acetabulum, a cup-shaped cavity in the pelvis. The labrum, a ring of tissue around the hip joint, and the surrounding ligaments help maintain hip stability (Figure 2). Between the acetabulum and the femoral head lies a smooth, glassy substance called cartilage, which acts as a frictionless cushion, enabling the smooth and constrained movement of the femoral head within the acetabular socket [29].

The hip joint, which endures significant daily stress, can become impaired due to pathological changes or injuries. Such dysfunction can lead to pain, reduced mobility, and considerable discomfort for the patient. Problems originating from either the acetabulum or the femoral bone are among the most common causes of hip joint diseases. For a comprehensive list of hip joint issues that can lead to severe disability, refer to [7,17], where the meaning of the medical terminology of the major diseases is briefly discussed. In some cases, to restore a patient to a more normal lifestyle, surgical intervention may be necessary, involving the replacement of the damaged hip joint with a prosthesis.

An interesting chronological reconstruction of the early attempts at hip arthroplasty from 1700 to 1950 is described in [30]. This work traces nearly three centuries of evolution in the treatment of hip arthritis, from rudimentary surgery to modern THA, which is considered one of the most successful surgical procedures ever developed. The article reviews the history of hip arthroplasty procedures that preceded Charnley’s THA, starting from early hip arthritis surgery in the 1800s, through interpositional hip arthroplasty in the early 1900s, up to the first hip replacements in the mid-1900s.

A comprehensive review of radiographic evaluations for hip arthroplasty, encompassing classifications using various types and techniques, terminology related to prosthetic designs and materials, surgical methods, and both initial and follow-up radiographic assessments, is detailed in [31]. Specifically, hip joint surgery techniques can be broadly categorized into two main groups: Partial Hip Arthroplasty (Hemiarthroplasty) and Total Hip Arthroplasty (THA). Hemiarthroplasty involves the partial replacement of hip components, typically the femoral head, without addressing the acetabulum. In contrast, THA involves the complete replacement of both the femoral head and the acetabular cup. THA’s remarkable success has earned it the designation of “the operation of the century” [32,33].

**Figure 3:**
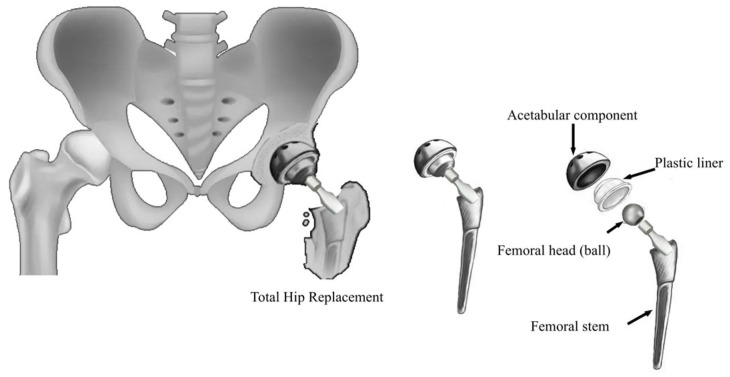
Individual components of a THR (**right**); assembled components forming the complete implant (**center**); the implant fitted into the hip joint (**left**).

Figure 3 illustrates the basic principle of THA. This procedure typically involves replacing the entire hip joint with a modular prosthesis typically consisting of three main components: a femoral stem, a femoral head, and an acetabular cup. Both the femoral stem and the acetabular cup can be secured to the bone using either cemented or uncemented techniques. In uncemented systems, the acetabular cup comprises an external metal shell and an internal liner. The femoral stem is inserted into the femur, providing support for the femoral head, which articulates smoothly with the liner. The liner, in turn, is housed within the shell, which is securely attached to the pelvic bone cavity [34].

The femoral stem is typically made from metallic materials, such as titanium alloys or stainless steel. The femoral head can be made of either ceramic or metal, with ceramic being preferred in some cases for its superior wear resistance. In cemented implants, the acetabular cup is a single polymer component, usually made of polyethylene. In cementless implants, the acetabular cup consists of two parts: a metallic acetabular shell and a liner. The liner, which articulates with the femoral head, can be made of either ceramic or polymer (commonly polyethylene). All materials used in hip prostheses are specifically designed to be biocompatible and minimize adverse reactions within the human body [34].

Charnley [35] revolutionized the treatment of arthritic hips by introducing the concept of low-friction arthroplasty, which marked a turning point in THR surgery. He significantly advanced the field by developing low-friction torque arthroplasty, using acrylic bone cement to securely fix components to living bone, and incorporating High-Density PolyEthylene (HDPE) as a durable bearing material. Charnley openly explains that his approach was derived from previous methods, particularly those of Smith–Petersen and Judet. The Smith–Petersen technique represented an advancement compared to earlier arthroplasty methods by introducing a membrane or tissue between the femoral head and its socket. Specifically, this approach involved placing a loose-fitting metal cup between the surgically prepared joint surfaces to improve joint function. In contrast, the Judet procedure focused on replacing the femoral head with a prosthesis featuring a smooth, metal hemispherical surface, aiming for enhanced articulation and stability. Building on these innovations, Charnley developed a THR system that included a press-fit plastic acetabular socket, a femoral component with a smooth metal head, and a cemented intramedullary stem. This design sought to achieve better stability and long-term integration of the implant.

Since then, despite early designs having a poor performance and frequent failures, continuous advancements in implant designs, biomaterials, surgical techniques, and the understanding of hip biomechanical restoration have significantly improved implant longevity and the clinical outcomes of THA over the past three decades. Thanks to their enhanced wear resistance and mechanical reliability, modern THA implants are now expected to last at least 25 to 30 years [36].

Recent advances in AM have opened new possibilities in the design of femoral stems, particularly in addressing long-standing issues such as aseptic loosening and stress shielding. The ability to fabricate implants with tailored porosity and complex geometries allows for better biomechanical compatibility with the surrounding bone [13,20]. Porous structures produced through AM techniques, such as Selective Laser Melting (SLM), enable a significant reduction in the elastic modulus of metallic implants, thus minimizing the stiffness mismatch with bone and promoting greater physiological load transfer [14,17]. This helps reduce stress shielding, a key factor in periprosthetic bone resorption. Moreover, porous AM surfaces offer an increased surface area and interconnectivity, enhancing osseointegration and mechanical interlocking, which are essential to improve implant stability and reduce the risk of aseptic loosening [12,19]. Design strategies that leverage AM include lattice-based architectures with optimized unit cell geometries, functionally graded porosity along the stem length, and targeted porous coatings in the metaphyseal region. These features support localized bone ingrowth and allow for a tailored mechanical performance along different regions of the stem [17,21]. Such innovations are particularly beneficial for uncemented implants and younger, more active patients, where biological fixation is preferred and long-term performance is critical [20].

Aseptic loosening remains the leading cause of implant failure and revision surgeries, primarily due to insufficient long-term fixation and stress shielding-induced bone resorption. The novel stem designs discussed herein, particularly those incorporating porous structures enabled by AM, directly address these issues. By reducing the implant’s effective stiffness, these designs promote a more natural load distribution and help preserve periprosthetic bone [14,17]. Additionally, the interconnected porous networks enhance early-stage osseointegration and provide long-term biological anchorage, minimizing micromotion at the bone–implant interface, which is a known trigger for fibrous tissue formation and implant loosening [12,19]. Thus, these strategies contribute to improving the mechanical and biological integration of the implant, ultimately reducing the risk of aseptic loosening.

It should be noted that recent technological innovations have brought significant changes to prosthetic surgery. At the forefront of these advancements are Virtual Reality (VR), 3D printing with AM, and robot-assisted surgery. VR enhances preoperative planning and simulation, allowing surgeons to practice intricate procedures in advance. Three-dimensional printing enables the creation of implants custom-fitted to a patient’s specific anatomy, thereby improving the fit and functionality of the prosthesis. Robot-assisted surgery offers increased precision and control during operations, reducing the risk of human error and enhancing surgical outcomes. Collectively, these technological advancements lead to more effective and durable hip replacements, improving patient care and accelerating the recovery times. These developments are comprehensively explored in [37].

Finally, a detailed examination of the mechanics and performance characteristics of new hip implant designs with innovative geometries is provided in [38]. The review focuses on how poor stress distribution from the implants to the femur can result in stress shielding, bone loss, excessive micromotion, and, ultimately, aseptic loosening due to inflammatory responses. The paper explores recent advancements such as implants with porous mesh structures, FGM stems, femoral resurfacing, short stems, and collared stems. These designs aim to achieve a more even stress distribution and facilitate proper bone remodeling.

## 4. Longevity and Failure Mechanics of THA

**Figure 4:**
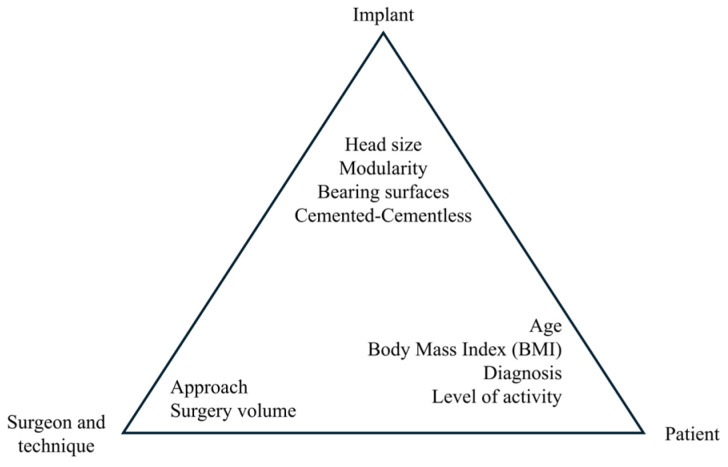
Factors contributing to failure modes in THA. Readapted from [19,20].

Although THA generally yields successful results, there has been a steady rise in revision rates in recent years [7]. This increase can be attributed to various factors, such as higher levels of physical activity, younger patients opting for the procedure, and a growing life expectancy in an aging global population [39]. The patterns of failure in THA have evolved over time. Understanding the mechanisms and timing of their occurrence can help reduce the need for revisions. The research [19,20] has identified three key groups of factors contributing to the failure of THA and leading to the need for revision (Figure 4): patient-related factors, issues stemming from inadequate surgeons and techniques [40], and implant-related factors [41]. Recognizing these factors, along with insights into the optimal timing for revisions and the identification of risk factors, facilitates better anticipation and management of potential issues. Applying this knowledge can enhance the overall outcomes of THA, improving both the longevity and effectiveness of the implant for patients.

**Figure 5:**
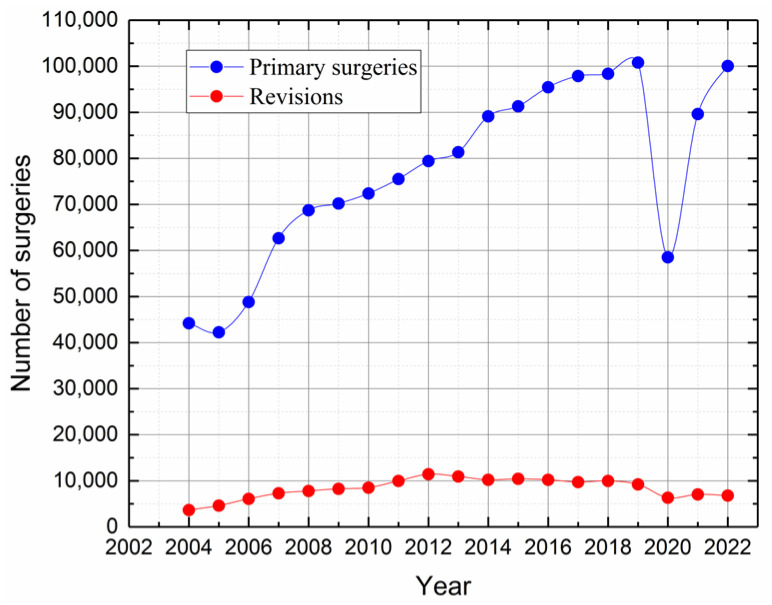
Number of primary and revision surgeries per year. Data are obtained from the NJR [21].

Since 2003, the NJR for England, Wales, and Northern Ireland has been compiling data on hip arthroplasties, and is currently overseeing one of the largest global repositories for hip replacement information. Patients who underwent surgery after the establishment of the registry have benefited from nearly 20 years of potential follow-up. According to the NJR’s 2023 report [21], between 1 April 2003, and 31 December 2022, approximately 1.45 million primary hip replacement surgeries (Figure 5) and over 140,000 revision surgeries were performed (around 9.7%).

The registry provides the percentages of primary hip replacements performed during the data collection period, categorized by type of fixation as well as by type of bearing material. The fixation methods for hip replacements include cemented, uncemented, hybrid, resurfacing, and reverse hybrid types. Cemented implants use bone cement to secure both the femoral stem and the acetabulum. Uncemented implants rely on press-fit and bone integration within the femur and acetabulum, which may be enhanced by additional methods, such as screw fixation. A resurfacing implant is used in hip resurfacing, where only the damaged surface of the femoral head is removed and capped with a metal cover, preserving more of the natural bone compared to a THR. Hybrid implants consist of a cemented femoral stem and an uncemented acetabulum, while reverse hybrid implants feature an uncemented femoral stem and a cemented acetabulum.

According to convention, the bearing material of the femoral head is listed before that of the acetabulum. For instance, a bearing combination described as “ceramic-on-polyethylene” indicates a ceramic femoral head and a polyethylene liner in the acetabular component.

**Figure 6:**
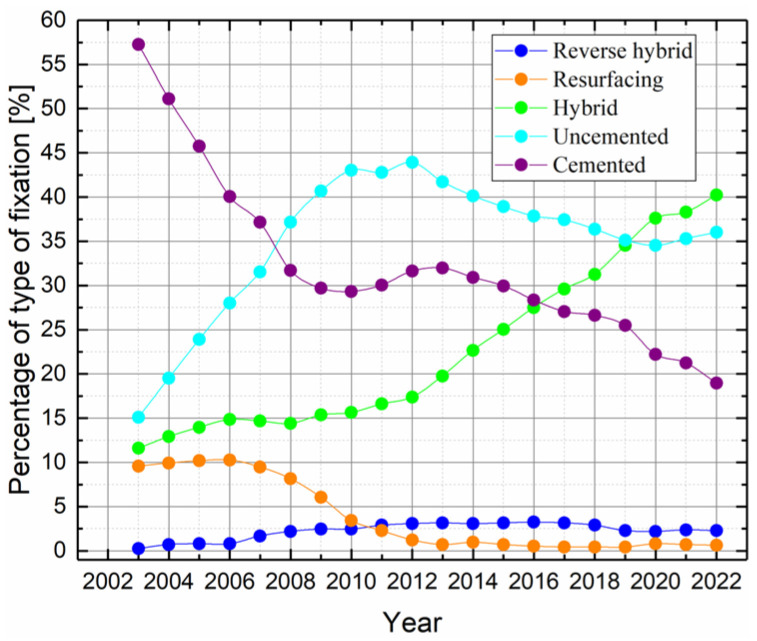
Trends in the percentages of primary procedures over time, by type of fixation method. Data are obtained from the NJR [21].

As shown in Table 1, hybrid implants are the most commonly used (40.3%), followed by uncemented implants (36.2%), cemented implants (19.1%), reverse hybrid implants (2.3%), and resurfacing implants (0.7%), and the remaining 1.4% represent unclassified cases. Figure 6 illustrates the trends over the years for each type of fixation. The figure indicates a recent decrease in the use of resurfacing and cemented fixation, with a corresponding increase in hybrid and uncemented fixation, while reverse hybrid fixation has remained relatively stable.

**Figure 7:**
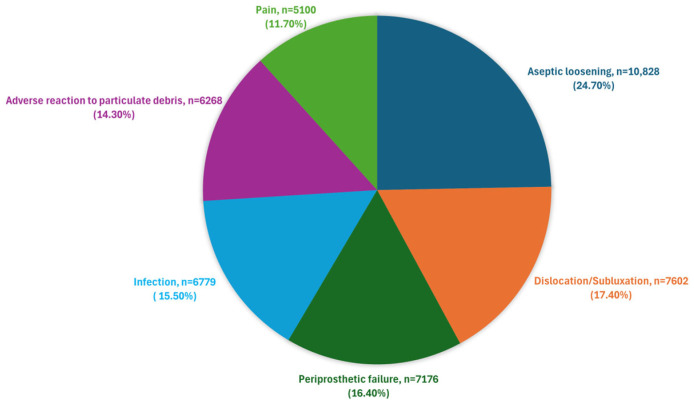
Percentage of total revisions for the most common indications. Data are obtained from the NJR [21].

Concerning the revisions, overall, only 43,682 (3%) of the 1,448,541 primary hip replacements had an associated first revision. Figure 7 shows a pie chart of total revisions for the most common indications reported in [21]. Along with these, there are a series of other, more specific indications listed in Table 2 in descending order according to the percentage of associated first revisions, which may be considered subcategories of the most common indications.

It should be noted that associating the issues listed with a single factor of THA failure is challenging. A closer analysis reveals that most issues are linked to multiple factors, suggesting that the interplay between these factors is crucial. For instance, the main indications were as follows:

1. Aseptic loosening [42] often arises from inadequate interaction between the implant and the surrounding bone. This issue can be worsened by cement fractures, which weaken the bond between the implant and bone, leading to micromotion and stress-related loosening. Discrepancies in the elastic modulus between implant materials and the surrounding bone further contribute to loosening, highlighting the need for materials with suitable mechanical properties and biocompatibility. A major factor in aseptic loosening is bone mass reduction due to stress shielding [43]. The significant stiffness difference between the prosthesis and cortical bone causes the implant to absorb most of the mechanical stress, leaving only a small portion of the load for the surrounding cortical bone. Consequently, the stress is unevenly distributed between the prosthesis and femur, reducing the mechanical load on the bone.
2. Dislocation or Instability [44] can result from a combination of factors, including improper alignment, faulty implant design, or inadequate surgical technique. Dislocation may occur due to a mismatch between the femoral head and the acetabular socket, leading to instability. Poor alignment or an incorrect size of the components can disrupt the natural biomechanics of the hip joint, increasing the risk of dislocation. Additionally, inadequate surgical technique or suboptimal implant design can compromise joint stability. Addressing these factors through precise surgical planning, careful component selection, and proper alignment can help minimize the risk of dislocation and ensure long-term joint stability.
3. Periprosthetic femoral fractures [45] can result from trauma or surgical errors, but patient-specific factors such as bone density and overall bone health also play a significant role. High-impact activities or falls can lead to fractures around the prosthesis, particularly in patients with compromised bone quality or those who have undergone multiple surgeries. Surgical errors, such as improper placement of the implant or inadequate fixation, can weaken the surrounding bone and increase the risk of fracture. Furthermore, bone quality around the implant can deteriorate over time due to stress shielding or osteopenia, making the area more susceptible to fractures. Effective prevention strategies include careful surgical planning, proper implant placement, and addressing bone health issues through preoperative assessments and postoperative management.
4. Infections [46] can result from non-sterile surgical techniques or complications during the procedure. Cemented implants are at risk if the adhesive cement is improperly applied or if there is a breach in sterility. The choice and effectiveness of the antimicrobial agents used in the cement are crucial in preventing infections, highlighting the importance of material quality and biocompatibility in infection control.
5. Adverse reactions to implant materials [47,48] are directly related to the materials used and their biocompatibility. The choice of implant materials affects how well the body accepts the implant and can influence the long-term outcomes.
6. Pain [49] experienced by the patient may result from a combination of surgical technique, implant adequacy, and individual patient response.

The more specific indications are as follows:

7. Implant malalignment [50] generally stems from errors in surgical technique or preoperative planning. Malalignment can lead to abnormal wear patterns and increased stress on certain parts of the implant, potentially accelerating wear and causing early failure. Precise alignment is critical to ensure even load distribution and proper joint mechanics, reducing the risk of complications such as dislocation, impingement, and irregular wear of the implant surfaces.
8. Lysis [51] is often due to an inflammatory reaction to implant debris, and is influenced by implant quality and design as well as surgical technique. The material properties of the implant, such as wear resistance and biocompatibility, are critical in mitigating inflammatory reactions.
9. Implant wear [52,53] is linked to the quality and material of the implant, affecting the longevity of the prosthetic components. The material’s durability, resistance to wear, and biocompatibility are decisive for maintaining implant function over time. Poor material quality can lead to increased wear particles, which can cause inflammatory reactions and contribute to osteolysis and aseptic loosening. Ensuring the use of high-quality, biocompatible materials helps to reduce wear rates, prevent adverse tissue reactions, and enhance the overall lifespan of the implant.
10. Implant fractures [54,55,56] can include fatigue fractures from repeated stress, impact fractures from trauma, and defects due to manufacturing flaws. The material’s resistance to fatigue and impact, as well as its biocompatibility, are essential in preventing such fractures.
11. Head/socket size mismatch [57] relates to implant design and selection during surgery. An improper match between the size of the femoral head and the acetabular socket can lead to increased wear, instability, and a higher risk of dislocation. Ensuring a proper fit is essential to maintain joint stability and function, as well as to minimize wear and prolong the lifespan of the implant.

According to NJR [21], failures are classified into two categories: early and late. Specifically, early failures occur soon after surgery and may be related to issues such as surgical complications or immediate implant problems; late failures, instead, develop over a longer period and are often associated with wear and tear, long-term implant issues, or changes in the surrounding bone or soft tissue. NJR provides insights into the revision rates for different failure mechanisms over time. Table 3 summarizes the trends and timings of the revision rates for various failure mechanisms in THA. It highlights the importance of monitoring different issues at various stages post-surgery to ensure timely and effective intervention.

## 5. Material Role, Selection Criteria, and Methods in THA

### 5.1. Material Significance in THA Failure

Based on the considerations outlined in the previous section, it is evident that material plays a pivotal role in reducing the likelihood of THA failure. Specifically, the following points emerge:

- Differences in elastic properties between implant materials and the surrounding bone can lead to stress shielding and consequent aseptic loosening, one of the primary causes of prosthetic failure [43,58,59].
- While less common, the material’s resistance to toughness, impact, fatigue, and creep can have severe consequences for patients in the event of fractures. Therefore, preventing fractures caused by repetitive stress, trauma, or manufacturing defects is crucial [54,55,56].
- The quality of the implant material directly impacts the durability of the prosthetic components. Longevity, wear resistance, and biocompatibility are essential for ensuring long-term functionality. Poor-quality materials may generate more wear particles, leading to inflammatory responses, osteolysis, and aseptic loosening [18,52,53].
- The biocompatibility of implant materials is crucial in determining the body’s reaction to wear debris. High-quality, biocompatible materials can reduce wear rates, prevent adverse tissue reactions, and extend the implant’s overall lifespan [47,48,60].
- The selection and effectiveness of antimicrobial agents in the cement are crucial for preventing infections, underscoring the importance of material quality and biocompatibility [46,61].

Quantifying the material’s importance in minimizing THA failure can be complex, but data from the NJR report [21] and the percentages of different causes leading to implant revision provide valuable insights:

- Aseptic loosening (accounting for 24.7% of failures) is partially influenced by the material, alongside other factors.
- Adverse reactions to particulate debris (14.3%) are directly related to the material.
- Infections (15.5%) are largely caused by reactions to wear debris and partly influenced by the quality of antimicrobial materials.
- Implant wear (5.6%) and implant fracture (5.6%) are directly related to the material’s quality and strength.

Therefore, it can be concluded that approximately more than half of hip prosthesis revisions are influenced, directly or indirectly, by the quality and properties of the materials used. This highlights the importance of selecting and using high-quality, biocompatible, and durable materials to minimize the risk of THA failure.

### 5.2. Criteria for Material Selection

Since a hip prosthesis is designed to function within the human body, it is essential to use biomaterials for its construction. Biomaterials are substances specifically chosen to create artificial devices that can safely, reliably, and economically replace or support a part or function of the body while being accepted by the body [24]. These materials are synthetic or natural substances used to replace parts of living systems or to interact closely with living tissue.

The main classes of biomaterials used for the construction of hip prostheses are metals, polymers, ceramics, composites, and apatite [62]. These materials are employed either individually or in combination and are essential to all modern hip implant devices. Given the interaction and mutual influence between the organism and the prosthesis over time, selecting the most suitable materials for the implant is crucial to ensure long-term success.

**Figure 8:**
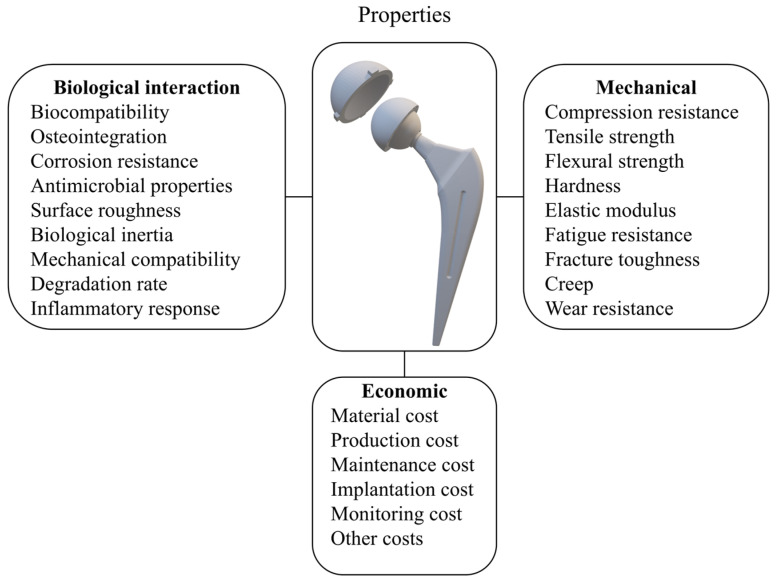
Criteria for material selection. Readapted from [20].

The primary selection criteria can be categorized into three main groups: biological interaction properties, mechanical properties and economic properties. As shown in Figure 8, each group includes a series of subcategories.

- **Biological interaction properties** reflect how materials interact with biological tissue and are crucial for the compatibility and success of implants. The most important ones are as follows:
  ○ Biocompatibility: the prosthesis must be in contact with the living system without producing any adverse effect. Mitigating the risks of immune responses is essential to ensure the success of the implant and prevent complications like implant rejection, cytotoxicity, and carcinogenesis [34,62,63].
  ○ Osteointegration: the artificial implant must fuse with the surrounding bone tissue for stability and long-term success. The aim is for bone to adhere to the implant surface, creating a strong bond resembling the natural connection, thus reducing the risk of failure. The osteointegration process involves the femoral stem and acetabular shell [64].
  ○ Corrosion resistance: the materials must withstand the corrosive effects of body fluids and the challenging conditions of the organism, such as prolonged exposure to heat and moisture, while maintaining structural integrity and preventing the release of metal ions [65].
  ○ Antimicrobial properties: the ability of the material to resist or inhibit microbial growth, reducing the risk of infections [61].
  ○ Surface roughness: the texture of the material’s surface, which can influence cell attachment and tissue integration [66,67].
  ○ Biological inertia: the property of remaining relatively neutral and non-reactive in the body.
  ○ Mechanical compatibility: matching mechanical properties with surrounding tissue to ensure proper load transfer. Additionally, the rate at which the material breaks down in the body should align with the tissue-healing process for optimal integration.
  ○ Degradation rate: the rate at which a material breaks down in the body, which should be compatible with the intended function and integration time of the implant.
  ○ Inflammatory response: the degree to which the material induces or mitigates inflammatory responses, affecting overall implant acceptance and function.
- **The mechanical properties** of the biomaterials used for implants include the following [68]:
  ○ Compression resistance: the material’s ability to withstand forces that tend to compress it without deforming or breaking.
  ○ Tensile strength: the material’s ability to resist forces that tend to pull or stretch it.
  ○ Flexural strength: the material’s capacity to resist deformation when bent.
  ○ Hardness: the material’s resistance to penetration or abrasion, affecting the durability and wear of the implant.
  ○ Elastic modulus: the stiffness of the femoral stem is crucial for load bearing. Since its stiffness impacts stress distribution, it should ideally have an elastic modulus comparable to that of the surrounding cortical bone (10 ÷ 30 GPa) to minimize stress shielding [58]. However, in practice, many femoral stems are made from materials with significantly higher elastic moduli, such as titanium alloys or cobalt-chromium alloys (100 ÷ 200 GPa). To counteract this mismatch, these implants are designed with specific geometries or surface treatments to reduce stress shielding. When the prosthesis is implanted, part of the load is transferred to the stem: the higher the stiffness of the implant, the more load it absorbs. This leads to a proportional reduction in the stress exerted on the bone, resulting in decreased bone density, which can accelerate osteoporosis progression and potentially cause aseptic loosening [59]. Emerging digital workflows and AM technologies—such as the 3D printing of porous titanium alloys—offer new possibilities for tailoring implant stiffness to match patient-specific biomechanics. By customizing internal architectures (e.g., graded porosity, lattice structures), it becomes feasible to engineer implants with site-specific elastic moduli that promote optimal load transfer and minimize stress shielding [59,69]. Moreover, the integration of biomechanical simulation tools with medical imaging enables the design of geometry- and load-optimized implants. These innovations are likely to drive the next generation of THA materials and designs, offering better personalization and long-term outcomes.
  ○ Fatigue resistance: the material’s ability to withstand repeated cyclic loads without fracture or failure [70].
  ○ Fracture toughness: the ability of the material to absorb energy and deform without breaking, which is essential for withstanding impacts and varying loads [71].
  ○ Creep: the slow and progressive deformation of the material under constant load over time, which can affect the long-term stability of the implant.
  ○ Wear Resistance: materials must resist mechanical wear from daily activities. Hence, components serving as bearing surfaces should possess appropriate coefficients of friction and minimize the generation of wear debris to prevent osteolysis and aseptic loosening. The release of toxic particles may induce adverse effects, such as allergic reactions and skin conditions [72,73]. Since surface wear is related to the types of materials that are mutually in contact, it might be interesting to analyze the types of bearing surfaces.

#### Bearing Surfaces

**Figure 9:**
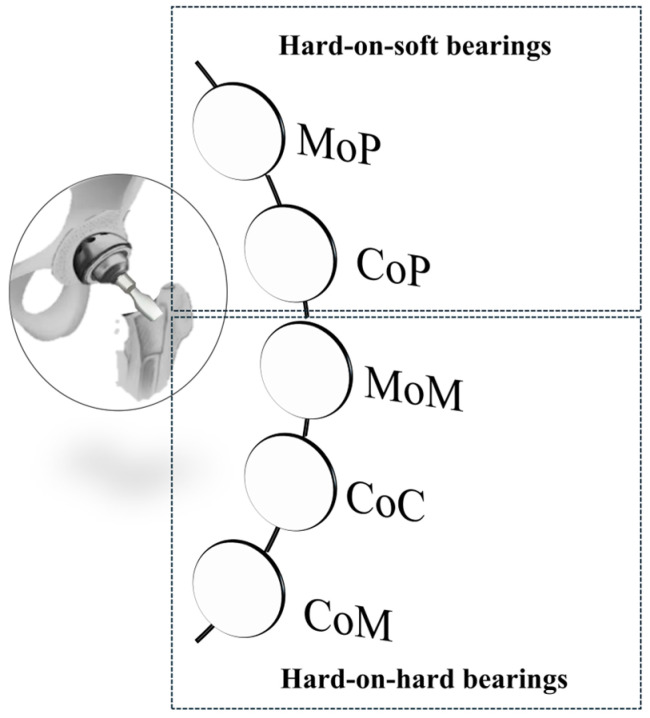
Combinations for bearing surfaces. Reprinted with permission from Ref. [35]. Copyright 1961 Elsevier.

Combinations of materials used for bearing surfaces fall into two main categories (Figure 9): hard-on-soft, indicating a metal or ceramic head coupled with a polymer liner, and hard-on-hard, in which both components are metal or ceramic. Identifying the most adequate coupling is necessary for ensuring the proper functioning and maximizing the longevity of the implant [35,74].

○ Metal-on-Polyethylene (MoP): since their initial introduction by Charnley in the 1960s, metal femoral heads and polyethylene acetabular liners have become the most commonly used materials in hip replacements. They are widely favored due to their proven safety and cost-effectiveness. Owing to its affordability and effective impact absorption, polyethylene and its derivatives are frequently employed in orthopedic applications, and are particularly suitable for elderly patients. Nevertheless, notable challenges arise from their susceptibility to high wear rates and osteolysis [60,75].
○ Ceramic-on-Polyethylene (CoP): these prosthetic systems feature a ceramic femoral head paired with a polyethylene acetabular liner. The ceramic head offers enhanced mechanical properties and superior resistance to scratching and deformation. Compared to other configurations, CoP systems demonstrate better wear characteristics thanks to the reduced friction coefficients. This is because worn ceramic heads retain smoother surfaces compared to their metal counterparts, particularly those made of cobalt–chromium alloys [60,75,76]. Despite its advantages, this approach has faced criticism due to its high cost and the potential risk of fractures in the ceramic femoral head;
○ Metal-on-Metal (MoM): during the late 1990s, MoM prostheses emerged as a popular trend among arthroplasty surgeons, particularly for younger, physically active patients. The literature data report lower osteolysis and wear rates, attributed to diminished particle diameters and friction values. However, the heightened levels of metal ions in the bloodstream may induce hypersensitivity reactions in soft tissues, leading to systemic complications, including pseudotumor formation. Consequently, MoM implants have been supplanted [60,75,77].
○ Ceramic-on-Ceramic (CoC): ceramic bearing surfaces combine high scratch resistance and low friction coefficients. Additionally, they release the smallest wear particles among tribological couples, reducing the risks of osteolysis and implant loosening. However, the limitations of CoC include squeaking, caused by head vibrations over worn surfaces, and ceramic fracture, a major complication arising from imperfections or misalignment of the acetabular cup. These risks, however, have been significantly reduced in newer generations of ceramic materials [60,75,78,79].
○ Ceramic-on-Metal (CoM): in CoM prostheses, a ceramic femoral head articulates with a metal acetabular liner. This design aims to eliminate the ion generation and catastrophic failure associated with MoM bearings, while also preventing fractures and squeaking, which can occur with CoC bearings [60,75]. This bearing configuration aimed to blend the durability of ceramics with the strength of metals [79]. Nonetheless, a controlled trial conducted by Higgins et al. [77] recently highlighted concerns regarding the overall safety of this combination. In comparing CoM with MoM systems, researchers found no significant differences in functional and radiological outcomes between the two groups. Initially, chromium ion levels were significantly lower in the CoM group for up to three years, but these levels increased after five years. The study noted some revisions in the CoM group due to metal debris. The researchers determined that CoM systems continued to produce metal ions, which suggested a different wear pattern compared to MoM systems. Consequently, they recommended further investigation into the safety and efficacy of CoM bearings.

- **Economic properties** reflect the financial and systemic impact of material selection, influencing the total cost of treatment and implant maintenance over the implant’s lifetime. As highlighted in the literature [80,81], material cost, manufacturing complexity, and revision-related expenses are increasingly important in decision-making. The following economic aspects are commonly considered:
  ○ Material cost: the price of the biomaterial or the implant itself, which can vary based on quality, the complexity of processing, and availability;
  ○ Production cost: the expenses associated with the production and manufacturing of the implant, including processing, the technologies used, and labor;
  ○ Maintenance cost: the costs anticipated for the preservation or replacement of the implant over time, including any necessary repairs or adjustments;
  ○ Implantation cost: the expenses related to the surgical procedure for implant placement, including medical staff fees, hospitalization, and equipment;
  ○ Monitoring cost: the costs associated with post-operative monitoring and follow-up visits to ensure the proper functioning of the implant;
  ○ Other costs: additional expenses that may arise related to the implant’s use and maintenance.

Balancing material performance with cost is crucial to ensure that high-quality implants remain accessible without sacrificing effectiveness. As emphasized in [80,81], an ideal biomaterial should have the following attributes: it must be biocompatible (innocuous to the host’s living cells), corrosion-resistant (able to endure the body’s chemical environment), low-friction (featuring a minimal friction coefficient for smooth movement and reduced wear over time), mechanically robust (capable of withstanding applied forces and preventing premature mechanical failures), and also cost-effective.

### 5.3. Methods for Material Selection

**Figure 10:**
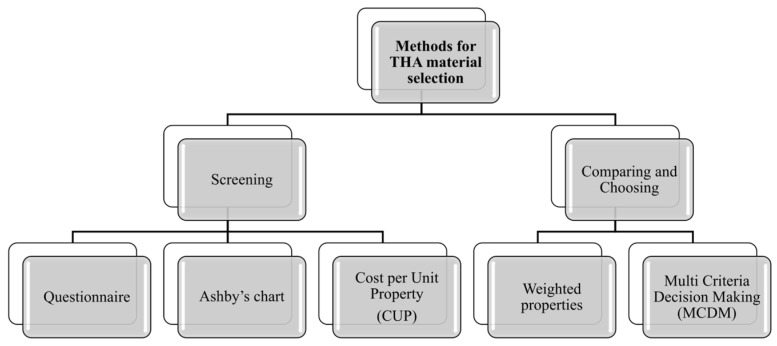
Classification of methods in material selection.

Selecting the most suitable materials in engineering design is acknowledged as a crucial part of the design process. Choosing from the thousands of materials that meet the various design requirements and possess the necessary characteristics is particularly challenging. Each material’s use necessitates a selection methodology, which can be viewed as a problem-solving activity. This task demands a decision-making process that leverages extensive expertise and engineering techniques. Throughout the years, researchers have developed various methods to enhance material selection strategies according to the desired properties. A systematic review summarizing many of these material selection methods is provided in [22]. The authors evaluated various strategies, classifying them into Screening and Choosing Materials methods, as well as approaches involving Artificial Intelligence (AI), Optimization and Mathematical techniques, and Fuzzy methods, detailing their respective advantages and drawbacks (Figure 10).

Below, the methodologies that can be used and have been used for material selection in THA will be briefly described.

Screening methods are an effective way to eliminate candidates that are not suitable for selection and to establish a potential set of appropriate alternatives from readily available large datasets that consist of material properties. Among them, the following deserve to be mentioned:

- Questionnaire method: this conventional method, which is the oldest and simplest approach to material selection, involves sourcing essential information about materials, including their physical properties and performance characteristics, from manufacturers, suppliers, and standards. The questionnaire method, although utilized in material selection, does not precisely fit within traditional screening methods. It acts as a tool for gathering information and directing the decision-making process through a set of structured questions designed to evaluate the compatibility and characteristics of materials for specific applications. This approach can be seen as a complementary methodology rather than an independent screening method. Referring to THA, consulting reports from national registries that collect data on surgical interventions and joint prostheses is highly beneficial for monitoring and enhancing treatment quality, as well as selecting appropriate materials. Numerous such registries exist globally, and some of the most significant ones are listed in Table 4.

**Figure 11:**
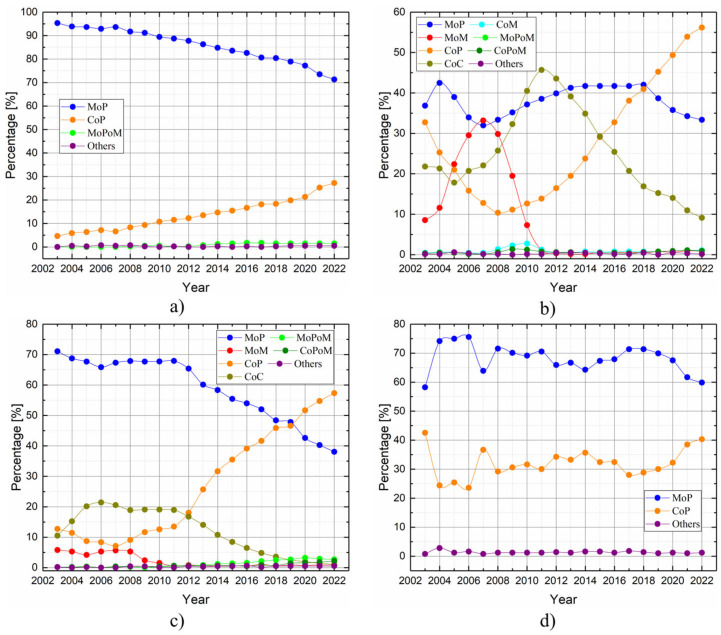
Temporal evolution of bearing surface materials concerning the type of THR fixation: (**a**) cemented primary hip replacement, (**b**) uncemented primary hip replacement, (**c**) hybrid primary hip replacement, and (**d**) reverse hybrid primary hip replacement. Data are obtained from the NJR [21].

For instance, when selecting materials for bearing surfaces, analyzing the temporal trends in the use of different material pairings can provide insight. Figure 11, reconstructed by assembling several graphs from the NJR [21], shows how the combinations of bearing surface materials have evolved over time concerning the type of THR fixation: (a) cemented, (b) uncemented, (c) hybrid, and (d) reverse hybrid. Since 2012, there has been a notable increase in the use of CoP bearings, accompanied by a decrease in CoC bearings. The most substantial variation in bearing material usage is seen in the uncemented fixation group.

**Figure 12:**
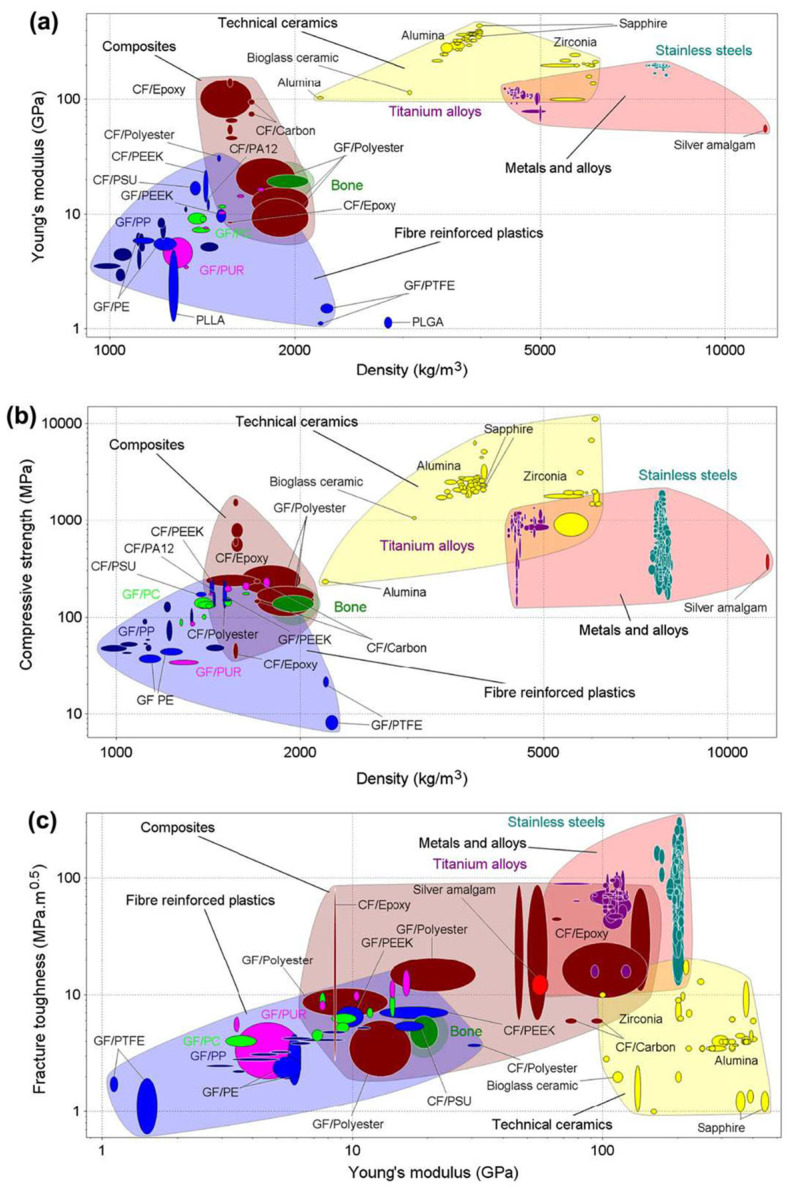
Comparison of (**a**) stiffness, (**b**) strength, and (**c**) fracture toughness for metals, technical ceramics, composites, and fiber-reinforced plastics with respect to the properties of bone. Reprinted with permission from Ref. [69]. Copyright 2011 Elsevier.

- Ashby’s chart method: this method allows for the easy and intuitive visualization and comparison of material properties. Ashby’s material selection technique utilizes scatter plots, known as Ashby Charts, to display material properties on cartesian axes. Each point on the chart represents a specific material, with its properties plotted accordingly. In a study by Scholz et al. [69], Ashby diagrams were used to compare the mechanical properties of materials used in orthopedic and prosthetic applications. These diagrams were essential for understanding the trade-offs between different material properties, such as stiffness, strength, and fracture toughness, in relation to bone. The authors compared the properties of bone with those of metals, ceramics, composites, and fiber-reinforced plastics (Figure 12). However, a notable limitation of these charts is that they can only consider two or three criteria simultaneously.

In addition to traditional paper-based procedures, computer-based materials selection methods have been developed. The first of these methods was developed from a collaboration between Michael Ashby, David Cebon, and Granta Design. Specifically, Ashby settled the theoretical basis and graphical tools for materials selection, David Dargie contributed to their practical application and dissemination, and Granta Design created the software CES Selector [82] and EduPack [83], which make these methodologies accessible and usable in both educational and industrial settings.

CES Selector was designed to be an advanced materials selection tool for use by engineers and designers in industrial settings. It is used to analyze, compare and select materials for a wide range of engineering applications. Conversely, EduPack was developed as an educational tool to help students and educators understand material properties, selection, and application in the context of engineering and design.

CES Selector was employed as the primary tool for selecting optimal materials for hip prostheses in [84]. The research team employed this software to define boundary limits based on the desired biomechanical performance of the hip prosthesis. CES Selector aided in identifying candidate materials that met these boundary limits, considering factors such as biocompatibility, biomechanical performance, and cost. After applying these criteria, CES Selector filtered out the materials that satisfied the required specifications.

- Cost per Unit Property (CUP) method: this is an economical approach to material selection based on the ratio of cost to a specific material property. This method is useful when the goal is to optimize cost in relation to a particular material characteristic, such as strength, stiffness, or density. Generally, it is suitable for an initial screening where one property stands out as the most critical service requirement. Moreover, a target cost is set to remove expensive materials. To the best of the authors’ knowledge, this method was not applied in the context of this study.

Choosing the right material requires extensive comparison, as multiple options are often available for a given application. After identifying potential materials, ranking strategies can be applied to evaluate and prioritize them, reducing the options to a manageable few. Among the Comparing and Choosing Methods, it is worth mentioning the appropriate methods found in the literature, such as Weighted Property Method (WPM) and Multi Criteria Decision Making (MCDM) technique.

- Weighted Properties Method (WPM): this method provides a systematic and quantitative approach to material selection, ensuring that all relevant properties are considered according to their relative importance. In the method, property “scoring” and weighting are typically performed by a multidisciplinary team of experts (biomedical engineers, orthopedic surgeons, materials scientists, and biomechanics experts). A typical application of this approach can be found in [34], where Hamidi et al. employed WPM to select the appropriate materials for hip prosthesis;
- Multi Criteria Decision Making (MDCM) technique: this approach aims to offer a well-founded recommendation to decision-makers by evaluating a finite set of alternatives against multiple criteria or objectives. It is particularly valuable in complex decision-making scenarios where trade-offs between different factors must be considered. By systematically comparing alternatives across various dimensions, MCDM helps ensure that the selected option aligns with the overall goals and constraints of the project. This method supports a balanced and comprehensive evaluation, leading to more informed and justifiable decisions [22].

In the field of material selection for THA, various MDCMs have been employed. Some examples are provided as follows:

○ Arian and Ashkan Hafezalkotob [25] developed an advanced method called MULTIMOORA (Multi Objective Optimization by Ratio Analysis plus the Full Multiplicative Form), which integrates target-based attributes and significant coefficients for material selection in biomedical applications. Using this method, they identified the best candidate materials for the femoral component of hip and knee prostheses. The MULTIMOORA method evaluates multiple criteria simultaneously, providing a comprehensive and robust decision-making framework that ensures optimal material performance and compatibility in medical implants.
○ Aherwar et al. [85] focused on the preliminary evaluation of new materials for the femoral head of the hip joint, applying an advanced MCDM technique to identify the best material. The combined use of the AHP (Analytic Hierarchy Process) and VIKOR (Vise Kriterijumska Optimizacija Kompromisno Resenjemeaning) allowed for a systematic and well-justified material selection, simultaneously considering multiple performance criteria.
○ In the study by Gul et al. [86], a method called Fuzzy PROMETHEE (Preference Ranking Organization Method for Enrichment Evaluation) is developed to effectively address material selection problems by incorporating the uncertainty and vagueness inherent in MCDM. This methodology is adaptable to various applications, not limited to biomedical fields such as THA.

- Artificial Intelligence (AI): to the best of the authors’ knowledge, there are no applications of AI for material selection in THA, but developments in this area are anticipated. The study by Jodeiri et al. [87] on estimating Pelvic Sagittal Inclination (PSI) using AI highlights a promising future application in material selection for THA. In fact, by optimizing the positioning of the acetabular component based on patient-specific anatomical data, AI can help to ensure that the selected materials are ideally suited to withstand the specific stresses and movements of the hip joint, thereby enhancing the performance and longevity of the implant. This approach, although currently limited to the acetabular component, demonstrates the potential for AI to refine and improve material selection processes in orthopedic implants.

Based on the statements made so far, the selection of materials can be carried out by applying one of the previously discussed selection methods. However, it is essential to remember that the choice of material should also consider the shape and conformation of the component, as well as the patient’s bone structure, as these factors can significantly influence the performance and longevity of the implant. Indeed, to guarantee major bone resorption, the selection of implant materials should consider the patient’s bone structure to ensure proper load distribution and minimize stress shielding. Additionally, different studies underscore the significance of tailoring material selection to the specific shape and load-bearing requirements of the implant component to optimize performance [88,89,90].

To complete the description of the materials that can be selected for the different parts of the hip prosthesis, Table 5 presents the results obtained by various authors. The table provides a comprehensive overview of the materials evaluated and chosen for different components of hip prostheses, based on various studies conducted by different authors over the years. Each row in the table summarizes key information, including the materials considered, the selection methods applied, and the specific properties optimized during the selection process, as follows:

- Various material options: the table highlights a variety of materials, including metals (e.g., titanium alloys and cobalt–chromium alloys), ceramics, polymers, and composites, with each evaluated for their suitability in hip prosthesis components.
- Selection methods: a range of selection methods have been applied, from Finite Element Analysis (FEA) and Ashby Charts to more advanced techniques like MULTIMOORA and CES Selector. This heterogeneity underscores the complexity of the material selection process and the need to consider multiple criteria.
- Properties to optimize: the optimization of properties such as biocompatibility, mechanical strength, corrosion resistance, and wear resistance is critical for ensuring the longevity and performance of the implant. Different studies emphasize different properties based on the specific requirements of the component being designed.
- Consistency across studies: some materials, like Ti-6Al-4V and Co-Cr alloys, are consistently identified as optimal choices for various components, reflecting their well-established performance in biomedical applications.

**Table 5:** Overview of the materials evaluated for different components of hip prostheses based on various studies.

Evaluated Materials	Selection Method	Hip Component	Properties to Optimize	Reference
**Co-Cr-Mo alloy** **Ti-6Al-4V** Stainless Steel 316L Pure titanium	AHP	General *	Fatigue strength Corrosion resistance Wear resistance Cost Biocompatibility Elastic modulus	[23]
Stainless steel 316, 317, 321, 347 Co-Cr alloy (castable) Co-Cr alloy (wrought) Pure titanium Ti-6Al-4V Epoxy-70% glass Epoxy-63% carbon Epoxy-62% aramid	MULTIMOORA	Hip femoral component	Elastic modulus Corrosion resistance Tensile strength Fatigue strength Toughness Wear resistance Density Cost Tissue tolerance	[25]
316L St Steel (cold worked) Co-28Cr-6Mo (cast, ASTM F75) **Ti-6Al-4V (hot forged, ASTM F620), Zirconia (ceramic, 3Y-TZP)** Alumina (ceramic, ZTA)	WPM	General *	Elastic modulus Yield strength Tensile strength Fatigue strength Corrosion rate Density	[34]
Fiber-reinforced composite	Ashby Diagrams	General *	Young’s modulus Compressive strength Fracture toughness	[69]
**NiTi alloy wire, annealed, austenitic** **NiTi alloy, austenitic** Polyarylamide (50% glass fiber) Stainless steel, austenitic, AISI 301 Stainless steel, austenitic, BioDur 108 Stainless steel, martensitic, AISI 410 **Pure titanium** **Ti-6Al-1V**	CES Selector integrated with FEA	General *	Density Yield strength Tensile strength Elastic modulus Hardness Biocompatibility Corrosion resistance	[84]
Co-30Cr-4Mo-1Ni with various tungsten percentages (0÷4 W%)	AHP integrated with VIKOR	Femoral head	Wear resistance Corrosion resistance Mechanical strength Density	[85]
Particulate composite Fiber composite Ti-6Al-4V Ti-6Al-4V (porous coat)	FEA	Femoral component of cemented THA	Stiffness Strength Fatigue limit Biocompatibility	[91]

* The term “General” refers to studies that do not specify a particular component of the hip prosthesis (such as the femoral head, femoral stem, or acetabular component).

## 6. Conclusions and Future Perspectives

In conclusion, this study highlights the critical role of material selection in the success and longevity of THA. The selection of materials for THA is a crucial factor that directly impacts the durability and success of the procedure. The choice of appropriate biomaterials—metals, ceramics, polymers, and composites—directly impacts the implant’s performance, wear resistance, and biocompatibility. Over time, advancements in material science have addressed common challenges, such as aseptic loosening, implant wear, and stress shielding, which are leading causes of prosthesis failure. Despite the growing body of research on the biomaterials used for THA, the literature remains limited by a lack of standardized evaluation criteria, inconsistent reporting of clinical outcomes, and relatively few long-term studies comparing novel materials. This review itself has some limitations: it does not include gray literature, is not registered under a systematic review protocol, and may be subject to selection bias despite our structured methodology and use of bibliometric tools.

Data from multiple arthroplasty registries confirm that approximately more than half of hip prosthesis revisions are linked to material-related factors, emphasizing the importance of selecting durable, high-quality biomaterials. Bearing surface combinations such as MoP and CoP remain the most used, but emerging materials and technologies, including FGMs and hybrid composites, are promising options for enhancing implant longevity and minimizing complications.

In parallel, the criteria and methods used for material selection were examined, including the use of MCDM techniques and WPM. These methods have proven effective in guiding the selection of the most suitable materials, as demonstrated by practical examples applied to optimize different prosthetic components. These applications illustrate how tailored approaches can address specific biomechanical needs and individual patient conditions. It is important to note that this paper represents Part 2 of a broader study. While the present work focuses on the criteria and methods used for selecting materials in THA, a comparative analysis of these biomaterials—including their clinical performance, complication rates, and revision outcomes—is thoroughly discussed in Part 1. Readers interested in specific data regarding materials with superior or inferior prognoses in THA are encouraged to refer to that first part for a complete overview.

Future research should focus on validating emerging biomaterials (e.g., bioactive composites, FGM, and surface-modified ceramics) through clinical trials and registry data. In parallel, greater attention should be directed toward patient-specific design technologies, leveraging digital workflows, biomechanical simulations, and AM to optimize implant fit, function, and long-term performance. Innovations such as 3D printing and the use of AI in preoperative planning may further refine the material selection process, leading to more personalized and durable THA solutions. Overall, continuous research and development in biomaterials are essential for improving clinical outcomes and ensuring long-term success in hip replacement surgeries.

## Figures and Tables

**Table 1:** Percentage of primary THR by implant type in 2022. According to the NJR [21], the total number of primary hip replacements in 2022 was 99043.

**Implant Type**	Hybrid	Uncemented	Cemented	Reverse Hybrid	Resurfacing	Unclassified
**Absolute value**	39,914	35,854	18,917	2278	693	1387
**Percentage**	40.3%	36.2%	19.1%	2.3%	0.7%	1.4%

**Table 2:** Clinical reasons for revision of THA and corresponding rates. Data are obtained from the NJR [21].

Indication for Revision	Percentage
Aseptic loosening	24.7%
Dislocation/subluxation	17.4%
Periprosthetic fracture	16.4%
Infection	15.5%
Adverse reaction to particulate debris	14.3%
Pain *	11.7%
**More specific indications**	
Malalignment	6.6%
Lysis	6.1%
Implant fracture	5.6%
Implant wear **	5.6%
Head/socket size mismatch	0.7%

* Pain was not usually cited alone but along with other indications. ** Implant wear refers to wear of the polyethylene component, wear of the acetabular component, or liner dissociation.

**Table 3:** Revision rates according to NJR [21].

Failure Mechanism	Early Failures (First Year)	Later Failures (Over Time)
Aseptic loosening	Fairly constant until five years	Steadily increases after five years
Pain	Increases over the first seven years	Declines after seven years
Subluxation/Dislocation	Higher in the first year	Decreases after the first year
Infection	Higher in the first year	Decreases after the first year
Malalignment	Higher in the first year	Decreases after the first year
Periprosthetic fracture	Highest in the first year, declines markedly	Begins to rise again at seven years
Adverse reaction to particulate debris	Increases until 15 years	Declines after 15 years
Lysis	-	Continues to rise with time

**Table 4:** Some of the most relevant arthroplasty registries.

Registry Name	Country	Website
National Joint Registry (NJR)	United Kingdom	The National Joint Registry-Working for patients, committed to excellence. (https://www.njrcentre.org.uk/, accessed on 5 May 2025)
German Arthroplasty Register (EPRD)	Germany	Home|EPRD (https://www.eprd.de/en, accessed on 5 May 2025)
American Joint Replacement Registry (AJRR)	United States	AAOS Registry Program-American Academy of Orthopaedic Surgeons (https://www.aaos.org/registries, accessed on 5 May 2025)
Australian Orthopedic Association National Joint Replacement Registry (AOANJRR)	Australia	Home-AOANJRR (https://aoanjrr.sahmri.com/, accessed on 5 May 2025)
Japanese Orthopedic Association National Registry (JOANR)	Japan	トップページ|JOANR (https://www.joanr.org/, accessed on 5 May 2025)
Canadian Joint Replacement Registry (CJRR)	Canada	Canadian Joint Replacement Registry (CJRR)|CIHI (https://www.cihi.ca/en/canadian-joint-replacement-registry-cjrr, accessed on 5 May 2025)
Swedish Arthroplasty Register (SAR)	Sweden	The Swedish Arthroplasty Register (https://sar.registercentrum.se/, accessed on 5 May 2025)
Dutch Arthroplasty Register (LROI)	Netherlands	LROI (https://www.lroi.nl/, accessed on 5 May 2025)
Brazilian Registry of Clinical Trials (REBEC)	Brazil	REBEC (https://ensaiosclinicos.gov.br/, accessed on 5 May 2025)
Korea Hip Replacement Surgery Registry (KHRSR)	South Korea	Korea Hip Replacement Surgery Registry|TrialScreen (https://app.trialscreen.org/trials/korea-hip-replacement-surgery-registry-study-nct06307145, accessed on 5 May 2025)
French Society for Orthopedic Surgery and Traumatology (SOFCOT)	France	Accueil|SOFCOT (https://www.sofcot.fr/, accessed on 5 May 2025)
Italian Arthroplasty Registry (RIAP)	Italy	RIAP-Italian Arthroplasty Registry (https://riap.iss.it/riap/en/, accessed on 5 May 2025)
Spanish National Hip Fracture Registry (RNFC)	Spain	rnfc.es (https://rnfc.es/, accessed on 5 May 2025)
South African Arthroplasty Society (SAAS)	South Africa	South African Arthroplasty Society|SAAS (https://arthroplastysociety.org.za/, accessed on 5 May 2025)
Indian Society of Hip & Knee Surgeons (ISHKS)	India	ISHKS (https://www.ishks.com/ijr.html, accessed on 5 May 2025)
New Zealand Orthopedic Association (NZOA)	New Zealand	NZOA Joint Registry|New Zealand Orthopaedic Association (https://www.nzoa.org.nz/nzoa-joint-registry, accessed on 5 May 2025)
Portuguese Arthroplasty Register (RPA)	Portugal	R.P.A-Home (http://www.rpa.spot.pt/?lang=en-GB, accessed on 5 May 2025)
Other arthroplasty registries	–	Arthroplasty Registries-NORE-EFORT (https://nore.efort.org/arthroplasty-registries, accessed on 5 May 2025)

## Data Availability

The original contributions presented in this study are included in the article. Further inquiries can be directed to the corresponding authors.

## Abbreviations

The following abbreviations are used in this manuscript:

AHP	Analytic Hierarchy Process
AI	Artificial Intelligence
AISI	American Iron and Steel Institute
AJRR	American Joint Replacement Registry
AM	Additive Manufacturing
AOANJRR	Australian Orthopedic Association National Joint Replacement Registry
ASTM	American Society for Testing and Materials
CES Selector	Cambridge Engineering Selector
CJRR	Canadian Joint Replacement Registry
CoC	Ceramic-on-Ceramic
CoM	Ceramic-on-Metal
CoP	Ceramic-on-Polyethylene
Co-Cr	Cobalt–Chromium alloy
Co-Cr-Mo	Cobalt–Chromo–Molybdenum alloy
CUP	Cost per Unit Property
EduPack	Name of an educational tool for material science
EPRD	German Arthroplasty Register
FDM	Fusion Deposition Modeling
FEA	Finite Element Analysis
FGMs	Functionally Graded Materials
HDPE	High-Density-PolyEthylene
ISHKS	Indian Society of Hip and Knee Surgeons
JOANR	Japanese Orthopedic Association National Registry
KHRSE	Korea Hip Replacement Surgery Registry
LROI	Dutch Arthroplasty Register
MCDM	Multi-Criteria Decision-Making
MoM	Metal-on-Metal
MoP	Metal-on-Polyethylene
MULTIMOORA	Multi Objective Optimization by Ratio Analysis plus the Full Multiplicative Form
NiTi	Nickel Titanium alloy
NJR	National Joint Registry
NZOA	New Zealand Orthopedic Association
OA	Osteoarthritis
PCL	Poly(ε-caprolactone)
PLA	Poly(lactic acid)
PROMETHEE	Preference Ranking Organization Method for Enrichment Evaluation
PSI	Pelvic Sagittal Inclination
REBEC	Brazilian Registry of Clinical Trials
RIAP	Italian Arthroplasty Registry
RFNC	Spanish National Hip Fracture Registry
RPA	Portuguese Arthroplasty Register
SAAS	South African Arthroplasty Society
SAR	Swedish Arthroplasty Register
SOFCOT	French Society for Orthopedic Surgery and Traumatology
ST Steel	Stainless Steel
THA	Total Hip Arthroplasty
THR	Total Hip Replacement
Ti-6Al-4V	Titanium alloy (6% Aluminum, 4% Vanadium)
VIKOR	Vise Kriterijumska Optimizacija Kompromisno Resenjemeaning
VR	Virtual Reality
WPM	Weighted Properties Methods
ZTA	Zirconia-Toughened Alumina

## References

[1] Crawford D.A., Adams J.B., Hobbs G.R., Morris M.J., Berend K.R., Lombardi A.V. (2021). Does Activity Level After Primary Total Hip Arthroplasty Affect Aseptic Survival?. Arthroplast. Today.

[2] Fan Z., Yan L., Liu H., Li X., Fan K., Liu Q., Li J.J., Wang B. (2023). The Prevalence of Hip Osteoarthritis: A Systematic Review and Meta-Analysis. Arthritis Res. Ther..

[3] This Common Form of Arthritis Is Projected to Impact Nearly 1 Billion People by 2050. https://www.health.com/billion-people-with-osteoarthritis-2050-7852650.

[4] American Joint Replacement Registry Surpasses 4.3 Million Captured Hip and Knee Arthroplasty Procedures. https://www.aaos.org/aaosnow/2024/dec/research/research04/.

[5] Number of Hip Replacements in OECD-Countries 2021. https://www.statista.com/statistics/283234/number-of-knee-replacements-in-selected-countries/.

[6] Tokgöz E. (2023). Total Hip Arthroplasty: Medical and Biomedical Engineering and Science Concepts.

[7] Pabinger C., Lothaller H., Portner N., Geissler A. (2018). Projections of Hip Arthroplasty in OECD Countries up to 2050. HIP Int..

[8] Erivan R., Villatte G., Dartus J., Reina N., Descamps S., Boisgard S. (2019). Progression and Projection for Hip Surgery in France, 2008–2070: Epidemiologic Study with Trend and Projection Analysis. Orthop. Traumatol. Surg. Res..

[9] Rasmussen M.B., El-Galaly A., Daugberg L., Nielsen P.T., Jakobsen T. (2022). Projection of Primary and Revision Hip Arthroplasty Surgery in Denmark from 2020 to 2050. Acta Orthop..

[10] Rupp M., Lau E., Kurtz S.M., Alt V. (2020). Projections of Primary TKA and THA in Germany From 2016 Through 2040. Clin. Orthop. Relat. Res..

[11] Nemes S., Gordon M., Rogmark C., Rolfson O. (2014). Projections of Total Hip Replacement in Sweden from 2013 to 2030. Acta Orthop..

[12] Shichman I., Askew N., Habibi A., Nherera L., Macaulay W., Seyler T., Schwarzkopf R. (2023). Projections and Epidemiology of Revision Hip and Knee Arthroplasty in the United States to 2040–2060. Arthroplast. Today.

[13] Cheung C.-L., Ang S.B., Chadha M., Chow E.S.-L., Chung Y.-S., Hew F.L., Jaisamrarn U., Ng H., Takeuchi Y., Wu C.-H. (2018). An Updated Hip Fracture Projection in Asia: The Asian Federation of Osteoporosis Societies Study. Osteoporos. Sarcopenia.

[14] Matsuoka H., Nanmo H., Nojiri S., Nagao M., Nishizaki Y. (2023). Projected Numbers of Knee and Hip Arthroplasties up to the Year 2030 in Japan. J. Orthop. Sci..

[15] Park J.-W., Won S.-H., Moon S.-Y., Lee Y.-K., Ha Y.-C., Koo K.-H. (2021). Burden and Future Projection of Revision Total Hip Arthroplasty in South Korea. BMC Musculoskelet. Disord..

[16] Inacio M.C.S., Graves S.E., Pratt N.L., Roughead E.E., Nemes S. (2017). Increase in Total Joint Arthroplasty Projected from 2014 to 2046 in Australia: A Conservative Local Model With International Implications. Clin. Orthop. Relat. Res..

[17] Bucholz R.W. (2014). Indications, Techniques and Results of Total Hip Replacement in the United States. Rev. Médica Clínica Las Condes.

[18] Evans J.T., Evans J.P., Walker R.W., Blom A.W., Whitehouse M.R., Sayers A. (2019). How Long Does a Hip Replacement Last? A Systematic Review and Meta-Analysis of Case Series and National Registry Reports with More than 15 Years of Follow-Up. Lancet.

[19] Kenney C., Dick S., Lea J., Liu J., Ebraheim N.A. (2019). A Systematic Review of the Causes of Failure of Revision Total Hip Arthroplasty. J. Orthop..

[20] Boubaker O. (2023). Medical and Healthcare Robotics: New Paradigms and Recent Advances.

[21] Achakri H., Ben-Shlomo Y., Blom A., Boulton C., Bridgens J., Brittain R., Clark E., Dawson-Bowling S., Deere K., Esler C. (2022). The National Joint Registry 20th Annual Report 2023.

[22] Rahim A.A., Musa S.N., Ramesh S., Lim M.K. (2020). A Systematic Review on Material Selection Methods. Proc. Inst. Mech. Eng. Part L J. Mater. Des. Appl..

[23] Çelik İ., Eroğlu H. (2017). Selection Application of Material to Be Used in Hip Prosthesis Production with Analytic Hierarchy Process. Mater. Werkst..

[24] Poovathikkal S., Nair N. (2018). Biomaterials and Biocompatibility. World J. Pharm. Res..

[25] Hafezalkotob A., Hafezalkotob A. (2015). Comprehensive MULTIMOORA Method with Target-Based Attributes and Integrated Significant Coefficients for Materials Selection in Biomedical Applications. Mater. Des..

[26] Aria M., Cuccurullo C. (2017). Bibliometrix: An R-Tool for Comprehensive Science Mapping Analysis. J. Informetr..

[27] Rohatgi, Ankit WebPlotDigitizer—Copyright 2010–2024. https://apps.automeris.io/wpd4/.

[28] OriginLab—Origin and OriginPro—Data Analysis and Graphing Software. https://www.originlab.com/.

[29] Understanding the Hip Joint Anatomy and Hip Joint Related Pain. https://hippainhelp.com/understanding-the-hip-joint-anatomy-and-hip-joint-related-pain/.

[30] Gomez P.F., Morcuende J.A. (2005). Early Attempts at Hip Arthroplasty—1700s to 1950s. Iowa Orthop. J..

[31] Mulcahy H., Chew F.S. (2012). Current Concepts of Hip Arthroplasty for Radiologists: Part 1, Features and Radiographic Assessment. Am. J. Roentgenol..

[32] Learmonth I.D., Young C., Rorabeck C. (2007). The Operation of the Century: Total Hip Replacement. Lancet.

[33] Knight S.R., Aujla R., Biswas S.P. (2011). Total Hip Arthroplasty—Over 100 Years of Operative History. Orthop. Rev..

[34] Hamidi E., Fazeli A., Mat Yajid M.A., Che Sidik N.A. (2015). Materials Selection for Hip Prosthesis by the Method of Weighted Properties. J. Teknol..

[35] Charnley J. (1961). Arthroplasty of the Hip a New Operation. Lancet.

[36] Shon W.Y., Park B.-Y., R R.N., Park P.S., Im J.T., Yun H.H. (2019). Total Hip Arthroplasty: Past, Present, and Future. What Has Been Achieved?. Hip Pelvis.

[37] Fontalis A., Epinette J.-A., Thaler M., Zagra L., Khanduja V., Haddad F.S. (2021). Advances and Innovations in Total Hip Arthroplasty. SICOT-J..

[38] Soliman M.M., Islam M.T., Chowdhury M.E.H., Alqahtani A., Musharavati F., Alam T., Alshammari A.S., Misran N., Soliman M.S., Mahmud S. (2023). Advancement in Total Hip Implant: A Comprehensive Review of Mechanics and Performance Parameters across Diverse Novelties. J. Mater. Chem. B.

[39] Ulrich S.D., Seyler T.M., Bennett D., Delanois R.E., Saleh K.J., Thongtrangan I., Kuskowski M., Cheng E.Y., Sharkey P.F., Parvizi J. (2008). Total Hip Arthroplasties: What Are the Reasons for Revision?. Int. Orthop..

[40] Kobayashi N., Yukizawa Y. (2023). Causes of Failure after Total Hip Arthroplasty: A Narrative Review of Literatures. J. Jt. Surg. Res..

[41] Colic K., Sedmak A. (2016). The Current Approach to Research and Design of the Artificial Hip Prosthesis: A Review. Rheumatol. Orthop. Med..

[42] Feng X., Gu J., Zhou Y. (2022). Primary Total Hip Arthroplasty Failure: Aseptic Loosening Remains the Most Common Cause of Revision. Am. J. Transl. Res..

[43] Huiskes R., Weinans H., van Rietbergen B. (1992). The Relationship between Stress Shielding and Bone Resorption around Total Hip Stems and the Effects of Flexible Materials. Clin. Orthop. Relat. Res..

[44] Duplantier N.L., McCulloch P.C., Nho S.J., Mather R.C., Lewis B.D., Harris J.D. (2016). Hip Dislocation or Subluxation After Hip Arthroscopy: A Systematic Review. Arthrosc. J. Arthrosc. Relat. Surg..

[45] Van Den Kieboom J., Tirumala V., Xiong L., Klemt C., Kwon Y.-M. (2021). Periprosthetic Joint Infection Is the Main Reason for Failure in Patients Following Periprosthetic Fracture Treated with Revision Arthroplasty. Arch. Orthop. Trauma. Surg..

[46] Lenguerrand E., Whitehouse M.R., Beswick A.D., Kunutsor S.K., Burston B., Porter M., Blom A.W. (2018). Risk Factors Associated with Revision for Prosthetic Joint Infection after Hip Replacement: A Prospective Observational Cohort Study. Lancet Infect. Dis..

[47] Miller R.A., Ro J.Y., Schwartz M.R. (2017). Adverse Tissue Reactions after Total Hip Arthroplasty. Ann. Diagn. Pathol..

[48] French J.M.R., Bramley P., Scattergood S., Sandiford N.A. (2021). Adverse Reaction to Metal Debris Due to Fretting Corrosion between the Acetabular Components of Modular Dual-Mobility Constructs in Total Hip Replacement: A Systematic Review and Meta-Analysis. EFORT Open Rev..

[49] Zhang B., Rao S., Mekkawy K.L., Rahman R., Sarfraz A., Hollifield L., Runge N., Oni J.K. (2023). Risk Factors for Pain after Total Hip Arthroplasty: A Systematic Review. Arthroplasty.

[50] Myers C.A., Laz P.J., Shelburne K.B., Judd D.L., Huff D.N., Winters J.D., Stevens-Lapsley J.E., Rullkoetter P.J. (2018). The Impact of Hip Implant Alignment on Muscle and Joint Loading during Dynamic Activities. Clin. Biomech..

[51] Huddleston H.D. (1988). Femoral Lysis after Cemented Hip Arthroplasty. J. Arthroplast..

[52] Zhu Y., Chiu K., Tang W. (2001). Review Article: Polyethylene Wear and Osteolysis in Total Hip Arthroplasty. J. Orthop. Surg..

[53] Shahemi N., Liza S., Abbas A.A., Merican A. (2018). Long-Term Wear Failure Analysis of Uhmwpe Acetabular Cup in Total Hip Replacement. J. Mech. Behav. Biomed. Mater..

[54] Bauer T.W., Schils J. (1999). The Pathology of Total Joint Arthroplasty. Skelet. Radiol..

[55] Bäcker H.C., Wu C.H., Kienzle A., Perka C., Gwinner C. (2022). Mechanical Failure of Total Hip Arthroplasties and Associated Risk Factors. Arch. Orthop. Trauma. Surg..

[56] Babić M., Verić O., Božić Ž., Sušić A. (2019). Fracture Analysis of a Total Hip Prosthesis Based on Reverse Engineering. Eng. Fract. Mech..

[57] McTighe T. (2015). Modular Head Mismatch in THA. ReconRev.

[58] Sun C., Wang L., Kang J., Li D., Jin Z. (2018). Biomechanical Optimization of Elastic Modulus Distribution in Porous Femoral Stem for Artificial Hip Joints. J. Bionic Eng..

[59] Yamako G., Janssen D., Hanada S., Anijs T., Ochiai K., Totoribe K., Chosa E., Verdonschot N. (2017). Improving Stress Shielding Following Total Hip Arthroplasty by Using a Femoral Stem Made of β Type Ti-33.6Nb-4Sn with a Young’s Modulus Gradation. J. Biomech..

[60] Zagra L., Gallazzi E. (2018). Bearing Surfaces in Primary Total Hip Arthroplasty. EFORT Open Rev..

[61] Getzlaf M.A., Lewallen E.A., Kremers H.M., Jones D.L., Bonin C.A., Dudakovic A., Thaler R., Cohen R.C., Lewallen D.G., Van Wijnen A.J. (2016). Multi-disciplinary Antimicrobial Strategies for Improving Orthopaedic Implants to Prevent Prosthetic Joint Infections in Hip and Knee. J. Orthop. Res..

[62] Aherwar A., K Singh A., Patnaik A. (2015). Current and Future Biocompatibility Aspects of Biomaterials for Hip Prosthesis. AIMS Bioeng..

[63] Learmonth I.D. (2003). Biocompatibility: A Biomechanical and Biological Concept in Total Hip Replacement. Surg..

[64] McCutchen J.W., Collier J.P., Mayor M.B. (1990). Osseointegration of Titanium Implants in Total Hip Arthroplasty. Clin. Orthop. Relat. Res..

[65] Mueller U., Bormann T., Schroeder S., Renkawitz T., Kretzer J.P. (2022). Taper Corrosion in Total Hip Arthroplasty—How to Assess and Which Design Features Are Crucial?. J. Mech. Behav. Biomed. Mater..

[66] Cubillos P.O., Dos Santos V.O., Pizzolatti A.L.A., Moré A.D.O., Roesler C.R.M. (2021). Surface Finish of Total Hip Arthroplasty Implants: Are We Evaluating and Manufacturing Them Appropriately?. J. Test. Eval..

[67] Ghosh S., Abanteriba S. (2016). Status of Surface Modification Techniques for Artificial Hip Implants. Sci. Technol. Adv. Mater..

[68] Kenney S., Garner C. (2021). Mechanical Properties of Biomaterials Used in Total Hip and Knee Arthroplasty. https://shareok.org/server/api/core/bitstreams/b24a4cb8-76a3-4ad7-851e-d31090feb3cb/content.

[69] Scholz M.-S., Blanchfield J.P., Bloom L.D., Coburn B.H., Elkington M., Fuller J.D., Gilbert M.E., Muflahi S.A., Pernice M.F., Rae S.I. (2011). The Use of Composite Materials in Modern Orthopaedic Medicine and Prosthetic Devices: A Review. Compos. Sci. Technol..

[70] Corda J., Chethan K.N., Shenoy S., Shetty S., Bhat S., Zuber M. (2023). Fatigue Life Evaluation of Different Hip Implant Designs Using Finite Element Analysis. J. Appl. Eng. Sci..

[71] Choroszyński M., Choroszyński M.R., Skrzypek S.J. (2017). Biomaterials for Hip Implants—Important Considerations Relating to the Choice of Materials. Bio-Algorithms Med-Syst..

[72] Buford A., Goswami T. (2004). Review of Wear Mechanisms in Hip Implants: Paper I—General. Mater. Des..

[73] Ghadirinejad K., Day C.W., Milimonfared R., Taylor M., Solomon L.B., Hashemi R. (2023). Fretting Wear and Corrosion-Related Risk Factors in Total Hip Replacement: A Literature Review on Implant Retrieval Studies and National Joint Replacement Registry Reports. Prosthesis.

[74] Khalifa A.A., Bakr H.M. (2021). Updates in Biomaterials of Bearing Surfaces in Total Hip Arthroplasty. Arthroplasty.

[75] Seyyed Hosseinzadeh H.R., Eajazi A., Sina A., Fokter S. (2012). The Bearing Surfaces in Total Hip Arthroplasty—Options, Material Characteristics and Selection. Recent Advances in Arthroplasty.

[76] Kothiyal G.P., Srnivasan A.B., Kothiyal G.P., Srinivasan A. (2016). Trends in Biomaterials.

[77] Higgins J.E., Conn K.S., Britton J.M., Pesola M., Manninen M., Stranks G.J. (2020). Early Results of Our International, Multicenter, Multisurgeon, Double-Blinded, Prospective, Randomized, Controlled Trial Comparing Metal-on-Metal With Ceramic-on-Metal in Total Hip Arthroplasty. J. Arthroplast..

[78] Affatato S., Jaber S.A., Taddei P., Zivic F., Affatato S., Trajanovic M., Schnabelrauch M., Grujovic N., Choy K.L. (2018). Ceramics for Hip Joint Replacement. Biomaterials in Clinical Practice.

[79] Piconi C., Maccauro G., Muratori F., Brach Del Prever E. (2003). Alumina and Zirconia Ceramics in Joint Replacements. J. Appl. Biomater. Biomech..

[80] Kurtz S.M., Ong K.L., Schmier J., Mowat F., Saleh K., Dybvik E., Kärrholm J., Garellick G., Havelin L.I., Furnes O. (2007). Future Clinical and Economic Impact of Revision Total Hip and Knee Arthroplasty. J. Bone Jt. Surg..

[81] Bozic K.J., Kamath A.F., Ong K., Lau E., Kurtz S., Chan V., Vail T.P., Rubash H., Berry D.J. (2015). Comparative Epidemiology of Revision Arthroplasty: Failed THA Poses Greater Clinical and Economic Burdens Than Failed TKA. Clin. Orthop. Relat. Res..

[82] Ansys Granta Selector|Materials Selection Software. https://www.ansys.com/it-it/products/materials/granta-selector.

[83] Ansys Granta EduPack|Software for Materials Education. https://www.ansys.com/it-it/products/materials/granta-edupack.

[84] Şensoy A.T., Çolak M., Kaymaz I., Findik F. (2019). Optimal Material Selection for Total Hip Implant: A Finite Element Case Study. Arab. J. Sci. Eng..

[85] Aherwar A., Patnaik A., Bahraminasab M., Singh A. (2019). Preliminary Evaluations on Development of New Materials for Hip Joint Femoral Head. Proc. Inst. Mech. Eng. Part L J. Mater. Des. Appl..

[86] Gul M., Celik E., Gumus A.T., Guneri A.F. (2018). A Fuzzy Logic Based PROMETHEE Method for Material Selection Problems. Beni-Suef Univ. J. Basic. Appl. Sci..

[87] Jodeiri A., Zoroofi R.A., Hiasa Y., Takao M., Sugano N., Sato Y., Otake Y. (2020). Fully Automatic Estimation of Pelvic Sagittal Inclination from Anterior-Posterior Radiography Image Using Deep Learning Framework. Comput. Methods Programs Biomed..

[88] Chen Q., Thouas G. (2015). Biomaterials: A Basic Introduction.

[89] Geetha M., Singh A.K., Asokamani R., Gogia A.K. (2009). Ti Based Biomaterials, the Ultimate Choice for Orthopaedic Implants—A Review. Prog. Mater. Sci..

[90] Parthasarathy J., Starly B., Raman S., Christensen A. (2010). Mechanical Evaluation of Porous Titanium (Ti6Al4V) Structures with Electron Beam Melting (EBM). J. Mech. Behav. Biomed. Mater..

[91] Fagan M.J., Lee A.J.C. (1986). Material Selection in the Design of the Femoral Component of Cemented Total Hip Replacements. Clin. Mater..

